# A categorisation of problems and solutions to improve patient referrals from primary to specialty care

**DOI:** 10.1186/s12913-018-3745-y

**Published:** 2018-12-20

**Authors:** James Greenwood-Lee, Lauren Jewett, Linda Woodhouse, Deborah A. Marshall

**Affiliations:** 1 Centre for Science, Athabasca University, 6th Floor, 345 6 Avenue SE, Calgary, Alberta T2G 4V1 Canada; 2 Geography & Planning, University of Toronto, Sidney Smith Hall, Rm 594, 100 St George St., Toronto, Ontario M5S 3G3 Canada; 3 Faculty of Rehabilitation Medicine, University of Alberta, 3-10 Corbett Hall, 8205 114 Street, Edmonton, Alberta T6G 2G4 Canada; 4 Canada Research Chair, Health Services and Systems Research, Arthur J.E. Child Chair in Rheumatology Outcomes Research, Department of Community Health Sciences, University of Calgary, Calgary, Canada; 5 3C56 Health Research Innovation Centre (HRIC), 3280 Hospital Drive NW, Calgary, Alberta T2N 4Z6 Canada

**Keywords:** Health system, Chronic illness, Intervention, Primary care, Secondary care, Specialty care, Referral

## Abstract

**Background:**

Improving access to specialty care has been identified as a critical issue in the delivery of health services, especially given an increasing burden of chronic disease. Identifying and addressing problems that impact access to specialty care for patients referred to speciality care for non-emergent procedures and how these deficiencies can be managed via health system delivery interventions is important to improve care for patients with chronic conditions. However, the primary-specialty care interface is complex and may be impacted by a variety of potential health services delivery deficiencies; with an equal range of interventions developed to correct them. Consequently, the literature is also diverse and difficult to navigate. We present a narrative review to identify existing literature, and provide a conceptual map that categorizes problems at the primary-specialty care interface with linkages to corresponding interventions aimed at ensuring that patient transitions across the primary-specialty care interface are necessary, appropriate, timely and well communicated.

**Methods:**

We searched MEDLINE and EMBASE databases from January 1, 2005 until Dec 31, 2014, grey literature and reference lists to identify articles that report on interventions implemented to improve the primary-specialty care interface. Selected articles were categorized to describe: 1) the intervention context, including the deficiency addressed, and the objective of the intervention 2) intervention activities, and 3) intervention outcomes.

**Results:**

We identified 106 articles, producing four categories of health services delivery deficiencies based in: 1) clinical decision making; 2) information management; 3) the system level management of patient flows between primary and secondary care; and 4) quality-of-care monitoring. Interventions were divided into seven categories and fourteen sub-categories based on the deficiencies addressed and the intervention strategies used. Potential synergies and trade-offs among interventions are discussed. Little evidence exists regarding the synergistic and antagonistic interactions of alternative intervention strategies.

**Conclusion:**

The categorization acts as an aid in identifying why the primary-specialty care interface may be failing and which interventions may produce improvements. Overlap and interconnectedness between interventions creates potential synergies and conflicts among co-implemented interventions.

**Electronic supplementary material:**

The online version of this article (10.1186/s12913-018-3745-y) contains supplementary material, which is available to authorized users.

## Background

In response to the increasing prevalence of chronic conditions and the associated shift in the global burden of disease [1] there is pressure to improve chronic care [2–5]. New models for the delivery of care have been proposed, emphasising better integration between primary and specialty care, coupled with systems for better patient self-management. The goal is a patient centric system, easily navigable, with seamless transitions, that ensures patients receive appropriate services in a timely manner [6–8]. Achieving this goal requires reshaping of the health system through health services delivery interventions; transitioning from a compartmentalized system that is structured in terms of health care services, to an integrated system that is restructured in terms of patient focused chronic care pathways.

Improving access to specialty care, which includes any specialized medical services that can only be provided by a physician specialist, has been identified as an important system level issue, as patient outcomes may be compromised when disease management is delayed. However, accessibility is not easily decoupled from the broader need for the co-ordination of primary and speciality care [9] to ensure that patients are diagnosed and receive timely and effective treatment to manage their conditions, as early as possible. Access to specialty care requires that such services can be provided either locally or remotely. Here, we focus only on the former, as the latter case is deserving of focused consideration. Given that specialty care is locally available, the management of the primary-specialty care interface is important both at a patient level, as a determinant of health outcomes, experience, and satisfaction, and at a systems level as a determinant of patient flows as this interface is prone to inefficiencies [10].

The primary-specialty care interface centres on the referral. There are many reasons a patient might be referred to specialty care including diagnosis, management advice, and treatment beyond the scope of the primary care physician [11]. With growing demand for specialty care, but limited resources to meet demand, referral quality is increasingly important to ensure efficient patient flow across the primary-specialty care interface; referrals should be necessary, appropriate, timely, and well communicated [12]. In addition, the health system must support the referral process by maintaining efficient information exchange and patient flow, especially given the broader need for the co-ordination of primary and speciality care [9].

The primary-specialty care interface is complex [13], with a breadth of potential deficiencies impacting the patient journey through the primary-specialty care interface and an equal breadth of interventions. Previous reviews [10, 14, 15] have synthesized evidence on interventions to improve the primary-specialty care interface. However, while in practice, symptoms of a poorly functioning primary-specialty care interface may be easy to recognize, their causes may not be. In addition, the primary-specialty care interface may be impacted by a breadth of potential deficiencies; with an equal breadth of interventions developed to correct them. The complexity of the primary-specialty care interface requires consideration for both multitude of influencing factors and potential consequences of any given intervention, as well as the interactions amongst interventions, both synergistic and antagonistic [15]. Theory driven approaches are necessary [16]; approaches that consider mechanisms of causality [17, 18] by mapping out the deficiencies, why they arise, and which strategies can be used to intervene based on evidence showing effectiveness.

In this review we deconstruct the primary-specialty care interface to produce a conceptual map between deficiencies that impact access of patients with chronic conditions who are referred to specialty care for non-emergent procedures, interventions and subsequent impacts. The objective is to create an organizational structure that enables system deficiencies to be identified and linked to potential intervention strategies, while considering potential interactions amongst intervention strategies, both synergistic and antagonistic. We focus on the system perspective, where the objective is to optimize the system to improve access to specialty care for non-emergent patients. Consequently, the patient and provider experience are not captured explicitly in this context. The practical outcome is the creation of a resource for health care organizations seeking to optimize the primary care/specialty care interface to improve access to specialty care for non-emergent patients.

## Methods

The categorization was developed through an iterative two staged process.

### Stage 1: Narrative literature review

Narrative literature reviews provide a flexible methodology to collect, map and summarize current state of knowledge amongst diverse studies, where a key advantage of the narrative review is the ability to examine a wide breadth of literature and to address multiple research topics [19]. This is appropriate here, as we seek to understand a wide range of problems that impact the necessity, appropriateness, timeliness and communication of the referral, as well as how the health system supports the referral process by maintaining efficient information exchange and patient flow. It is important to note that an inherent trade-off with narrative reviews is possible subjectivity in study selection that potentially leads to biases and non-replicability. To maintain transparency, the search strategy is appended. However, the goal of our search is not to inform a comprehensive systematic review, but rather serves to capture a representative sample of the literature sufficient to inform our categorization [20]. An iterative search strategy was developed to capture interventions that address deficiencies at the primary-specialty care interface that impact patient access to specialty care. Given our broad focus on the primary-specialty care interface we sought to limit our search to ensure a manageable number of results. Specifically, we limited our search to peer-reviewed literature published over the most recent 10 year time frame (from January 1, 2005 until May 31, 2014, at time of search), indexed in the MEDLINE and EMBASE databases (see Additional file 1). Medline and EMBASE were selected due to their broad subject coverage including clinical care, public health, health policy development and health services research. Databases such as the CINAHL database, which is a database of nursing and allied health literature, were not included. A grey literature search was also conducted. Studies of interventions meeting all inclusion criteria were considered to be eligible for review: 1) in English 2) report on intervention to correct deficiencies that impact the necessity, appropriateness, timeliness and communication of patient transitions across the primary-specialty care interface, and 3) report on human subjects via primary data, secondary data (review articles) or data validated computer simulation of a health system. Articles were screened by two reviewers at three sequential levels: title, abstract and full text. Quality criteria were not used as our objective was to be inclusive. Selected articles were categorized to identify broad classes of interventions to improve the necessity, appropriateness, timeliness, and communication of referrals. This initial categorization was completed in December 2014. Following this initial categorization, we conducted targeted searches of MEDLINE and EMBASE databases to locate peer-reviewed literature up to December 31, 2014, and searched reference lists of included articles to identify additional relevant articles.

We captured pertinent information from each selected article using a data extraction form, including: specialty, intervention strategy, intervention objective, study design, methods, reported impacts, limitations, and conclusions. Quality of evidence was not scored. Data extractions were completed by a single reviewer and reviewed by a second.

### Stage 2: Categorization development

To develop our categorization we described the change process, beginning with contextualizing the need for change, and then detailing the mechanisms for change, including contextual factors which are associated or influence outcomes [21]. The purpose was to assimilate and categorize a broad range of interventions to both highlight their individual functions as well as their relationships. Our end-goal was to develop a categorization based on the broad types of deficiencies that are observed at the primary-specialty care interface. The practical difficulty encountered was that much of the reviewed literature describes the causal mechanisms through which the intervention operates with a forward focus; providing explanations of how the intervention generates change via the intervention’s actions. Explanations and evidence as to why the performance of the primary-specialty care interface is failing were not always provided. As such, to achieve our objective, backwards extrapolation was used to identify deficiencies in the primary-specialty care interface and their causes based on the form of intervention and its actions. The process proceeded through four steps.

In the first step the intervention objectives and activities were recorded as reported in the reviewed articles. Accordingly, we described: 1) the context and the objective of the intervention; 2) intervention activities; and 3) intervention outcomes. In the second step, root causes were extrapolated by considering each intervention action in the context of its objective and identifying the root causes the identified action served to remedy. The data extracted from the included papers were then synthesized as themes and categorized with a focus on high level deficiencies and their causes thereby creating the categorization scheme [20]. In step three, the previously recorded intervention objectives and activities were remapped within the new categorization scheme. In the final step, a summary of reported impacts were linked to the intervention activities and we finished by discussing potential synergies and conflicts among intervention strategies. Although the above process is presented as linear, in practice the four steps were iterative allowing the categorization to evolve to its final form.

## Results

### Stage 1: Narrative literature review

**Fig. 1:**
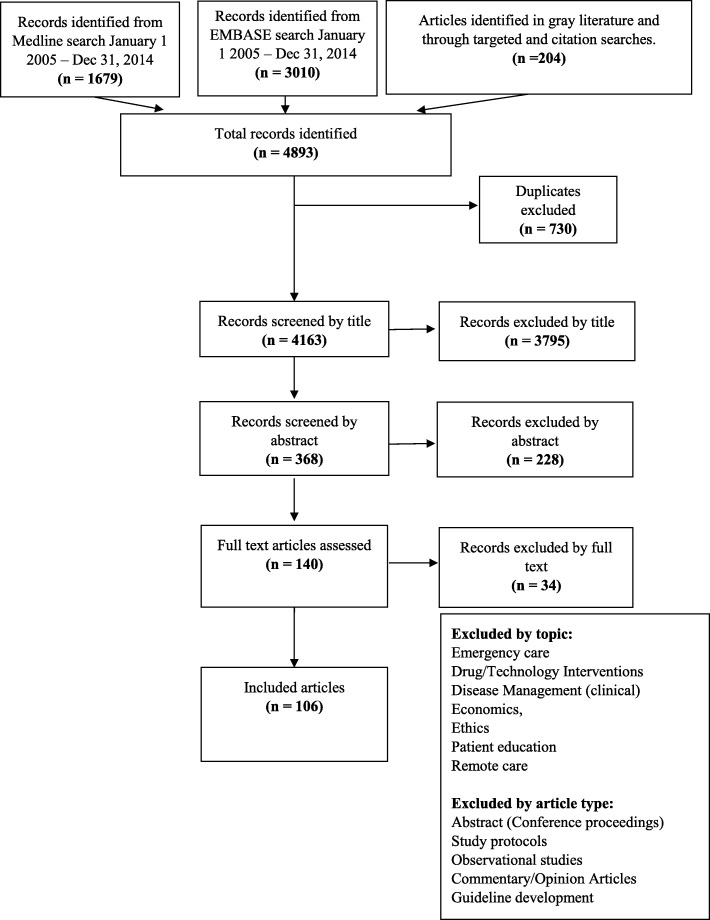
PRISMA diagram of included articles

The results of the literature search are presented in Fig. 1 (PRISMA diagram). The search returned 4893 records (Medline: 1679, Embase 3010, Articles Identified through targeted and citation searches 204). In total, 4787 records were identified as not meeting the inclusion criteria based on their title. Abstracts were reviewed in the remaining 368 articles. In total, 228 articles were excluded based on the information found in the abstract. 140 articles were extracted and reviewed in full text, with 106 of those included in this review.

Reviewed articles included systematic reviews, data validated computer simulations and empirical studies ranging from quality improvement reports, to time series analyses, and randomized controlled trials. Given the wide range of studies included in this review, the quality of the evidence reported varies among the reviewed articles. As previously noted, quality of evidence was not formally assessed here, as our objective is to provide an organizational structure that enables system deficiencies to be identified and linked to potential intervention strategies. Once the categories of intervention strategies are identified, evidence demonstrating intervention effectiveness can be reviewed as a next step. In particular, it will be important to determine if available evidence for a given category of intervention is highly context specific, or if there is a broad base of evidence that demonstrates the effectiveness of the intervention in variety of health services delivery applications and contexts.

### Stage 2: Categorization

**Fig. 2:**
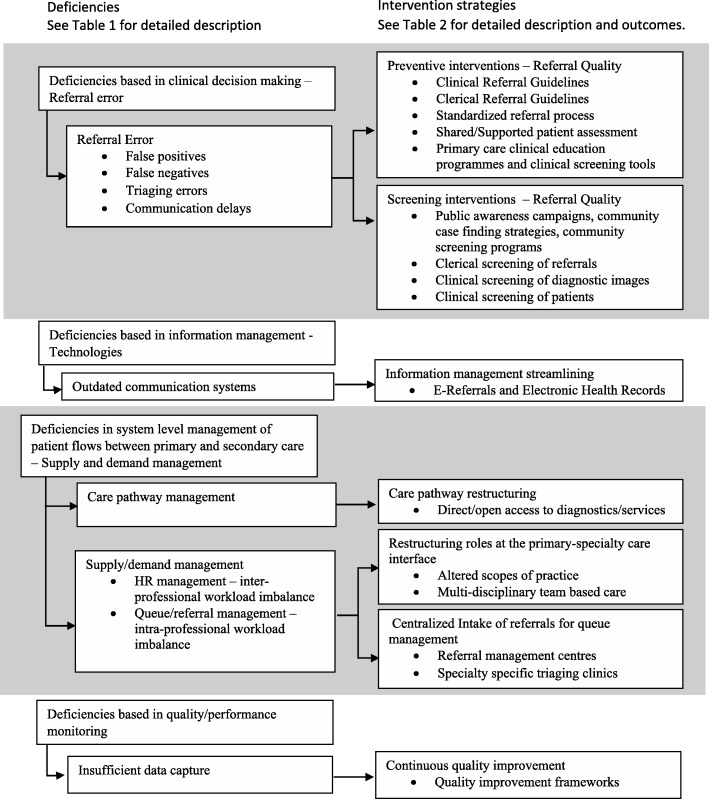
Simplified categorization linking deficiencies at the primary-specialty care interface to interventions

Four categories of health services delivery deficiencies were identified: deficiencies based in 1) clinical decision making, 2) information management, 3) the system level management of patient flows between primary and secondary care, 4) quality-of-care monitoring. Each is detailed below and mapped to intervention strategies, and reported impacts. Interventions were divided into seven categories and fourteen sub-categories based on the deficiencies addressed and the intervention strategies used. Figure 2 presents an overview of the categorization, with details captured in Tables 1 and 2.

**Table 1:** Deficiencies identified at the primary-specialty care interface

Intervention Context Identified deficiency in Health services delivery •Impact of deficiency •Intervention types and objectives			
D.1. Deficiencies based in clinical decision making – Referral error			
Deficiency	Sub-types and root cause	Impact of deficiency	Intervention types and Objectives (see Table 1B)
Referral errors as a natural consequence of diagnosis as a probabilistic process.	1. Unnecessary and inappropriate referrals (False positives) - Referral is unnecessary or inappropriate.	Poor patient experience, delay in receipt of diagnosis/care, compromised patient outcome. Impeded patient flows at the primary-specialty care interface and decreased efficiency of the health system. Potential impacts include: access delays, increased short term costs, increased long term costs as a result of a higher proportion of patients with severe conditions	Preventive interventions to reduce the occurrence of Type I errors, Type II errors, triaging errors and communication delays Screening interventions – to detect Type I errors, Type II errors, triaging errors and communication delays that have occurred.
	2. Delayed referrals (False negatives) - Referral is warranted, but not made.		
	3. Triaging Errors - Improper prioritization of patients based on urgency.		
	4. Communication delays - Referrals are delayed due to missing information and/or diagnostics.		
D.2. Deficiencies based in information management- Technologies			
Deficiency	Root cause	Impact of deficiency	Intervention types and Objectives (see Table 1B)
Delays due to outdated communication systems	Referrals and subsequent correspondence, forwarding of diagnostics, etc. via standard mail or fax.	Poor patient experience, delay in receipt of diagnosis/care, compromised patient outcome.	Electronic referral systems (e-referrals) and electronic medical records (EMRs) to expedite information sharing
D.3. Deficiencies based in system level management of patient flows between primary and secondary care – Supply and demand management			
Deficiency	Root cause	Impact of deficiency	Intervention types and Objectives (see Table 1B)
Delays due to care pathway structure and management	1. Care pathway management Inefficient care pathway structure and resource use hinders patient flows.	Poor patient experience, delay in receipt of diagnosis/care, compromised patient outcome. Impeded patient flows at the primary-specialty care interface and decreased efficiency of the health system. Potential impacts include: access delays, increased short term costs, increased long term costs as a result of a higher proportion of patients with severe conditions	Care pathway management - streamlining
Delays due to human resource management	2. Role management (Inter-professional workload imbalance) Creation of bottlenecks within the care pathway as a result of insufficient specialty care providers (Short term and sustained).		Human resource management - Scope of practice restructuring at primary-specialty care interface to increase supply of services
Delays due to queue/referral management	3. Queue/Referral management Intra-professional workload imbalance Disproportionate allocation of referrals amongst specialists producing an imbalance in specialist utilizations.		Queue/referral management - Centralized intake of referrals to improve access to specialty care.
D.4. Deficiencies based in quality/performance monitoring			
Deficiency	Root cause	Impact of deficiency	Intervention types and Objectives (see Table 1B)
Inability for performance improvement due to Insufficient data capture	Lack of a measurement framework to adequately track system performance	Decreased efficacy of operational decision making	Continuous quality improvement frameworks

**Table 2:** Intervention strategies and outcomes

Core intervention strategies Identified Health services delivery interventions •Strategy, objective, target population, Target impact, Reported Impacts				
	I.1. Interventions to reduce referral error			
	1. Preventive interventions to reduce the occurrence of false positives, false negatives, triaging errors and communication delays.			
Strategy	Objective	Target population	Target impact	Reported impact
Referral Guidelines	Clinical Guidelines: guidance on necessity, appropriateness and timeliness of referrals. Clerical guidance: guidance on completeness of referrals.	Referring physicians	Improve referral decisions: reduce false positives (unnecessary and inappropriate referrals), reduce false negatives (delayed referrals). Improve the communication of the referral.	Passive introduction of guidelines produces little change [36–47]. Guidelines with feedback may be effective [43, 53–56]. Coordinated introduction of guidelines, with standardized referral process can improve referral appropriateness and potentially lower wait times [64–70].
Standardized referral process	Set standards for referral quality. Aids include: standard referral forms, criteria checklists, diagnostic checklists, scoring systems.			
Shared/Supported patient assessment	Improved sensitivity/specificity of referral decision through decision support via professional networks: primary care groups, and multidisciplinary groups.	Referring physicians and allied health workers		Improved referral appropriateness while decision support is available [14, 58–61].
Primary care clinical education programmes and clinical screening tools	Improve sensitivity/specificity of referral decision through improved primary care disease specific knowledge and diagnostic screening tools	Referring physicians		Primary care education programmes and clinical screening tools. Reduction in number of delayed referrals, and improved referral appropriateness [48, 49, 64–70].
	2. Screening interventions – to detect false positives, false negatives, triaging errors and communication delays.			
Strategy	Objective	Target population	Target impact	Reported impact
Public awareness campaigns, community case finding strategies, community screening programs	Improved public awareness as to when to seek care with primary care provider, or direct public outreach.	Patient population.	Improved identification of patients in need of specialist care and reduction in number of delayed referrals	Improved identification of patients in need of specialist care and reduction in number of delayed referrals [87, 88].
Clerical screening of referrals	Screening of incoming referrals, to ensure referral completeness and triage referrals based on urgency.	Specialty care referral administration	Identify incomplete referrals, and request missing information from referring physician. Reduce scheduling of incomplete referrals.	Reduction of incomplete referrals being scheduled [75]. Improved triaging of referrals based on urgency [77–80].
Clinical screening of referrals	Confirm necessity and appropriateness of referral based on patient’s diagnostic images/test results that are requisite for the referral. Triage appropriate referrals.	Specialists/consultants	Identify and redirect false positives (unnecessary and inappropriate referrals), triage referrals based on urgency as indicated by diagnostic images/test results.	Redirection of unnecessary/ inappropriate referrals [10, 76].
Clinical screening of patients	Confirm necessity and appropriateness of referral based on clinical assessment of the patient. Identify appropriate care alternatives when specialist care is not required (e.g. conservative management for OA not requiring arthroplasty). Triage appropriate referrals.	Specialists/consultants, referring physicians and allied health workers	Identify and redirect false positives (unnecessary and inappropriate referrals), triage referrals based on urgency as indicated by clinical assessment.	Redirection of unnecessary/ inappropriate referrals
	I.2. Interventions to improve information management- Information technologies			
	Electronic referral systems (e-referrals) and electronic medical records (EMRs) to expedite information sharing			
Strategy	Objective	Target population	Target impact	Reported impact
Electronic referral systems and Electronic Medical records	Provide a standardized electronic referral process that ensures completeness of relevant information and diagnostics with the referral, and timely communication.	System level change affecting Primary care through specialty care.	Improve efficiency of referral submission. Reduce wait time between the time the referral is made and the time it is received and appointment is scheduled.	Electronic referral systems and electronic health records Improved workflow efficiency from referral submission, to screening and scheduling resulting from higher quality referral, improved access to diagnostics, and efficient communications [28, 29, 95]. Improved necessity/appropriateness of referrals due improved referral quality and enabling improved clerical/clinical screening of referrals [48, 92–94].
	I.3 Interventions to improve system level patient flows between primary and secondary care – Supply and demand management			
	1. Care pathway management - streamlining			
Strategy	Objective	Target population	Target impact	Reported impact
Direct/open access to diagnostics and specialty services	Provide direct, or open access, to specialty services by eliminating one or more steps along the traditional referral pathway.	System level change affecting primary care through specialty care.	Improved access to diagnostics, and specialty care.	Improved access to diagnostics and or specialty services, reduced waiting times [97–104].
	2. Human resource management - Scope of practice restructuring at primary-specialty care interface to increase supply of service			
Strategy	Objective	Target population	Target impact	Reported impact
Altered scopes of practice	Increased focus on specialty services by specialists supported with expanded scope of practice by non-specialist care providers.	Specialists and non-specialist care providers such as nurse practitioners, allied care and primary care.	Improved access to specialists via creation of capacity through alternative service delivery methods	Nurse led clinics/services: Reduced workload for physicians. Increased attention and timely care to patients. Evidence has shown nurse-led clinics can provide equivalent care with no greater risk of poorer outcomes [113, 114, 116] Similar outcomes reported for Primary care physicians with extended roles [119, 120].
Multi-disciplinary team based care	Coordinated care providing patient improved access to a broad range of complimentary care services.	Specialists and non-specialist care providers such as nurse practitioners, allied care and primary care.	Improved Quality of care. Improved access to specialists via creation of capacity through alternative service delivery methods.	Multi-disciplinary team based care: Better patient access to a wide range, but increasingly specialized health care services that may be required for effective disease management [123, 124]. Evidence regarding the cost-effectiveness of multidisciplinary team based care is lacking, and further studies are required [148].
	3. Queue/referral management - Centralized intake of referrals to improve access to specialty care.			
Strategy	Objective	Target population	Target impact	Reported impact
Referral management centres	Pooled intake and subsequent scheduling of all incoming referrals for all specialties	System level intervention, requiring critical buy-in from referring physicians and specialists.	Decreased wait times.	Systematic evaluations of referral management centres are needed [73].
Specialty specific triaging clinics	Pooled intake and subsequent scheduling of all incoming referrals for specific specialty (or sub-specialty)	System level intervention, requiring critical buy-in from referring physicians and specialists.	Decreased wait times.	Improved referral processes, improved patient triaging, improved appropriateness, improved wait times to specialist consult [58, 121, 132–134], unchanged wait times to downstream benchmarks such as surgery [135, 136], provider satisfaction may be low [135].
	I.4 Interventions to monitor and improve quality and/or performance			
	Continuous quality improvement			
Strategy	Objective	Target population	Target impact	Reported impact
Quality/performance improvement frameworks	Provide capacity for strategic decision making using information that relates operational and management decisions to their outcomes	System, Hospital, Clinic level targets	Improve strategic decision making, and management of operations.	Performance measures of referral processes and outcomes including the adequacy and appropriateness of coordination of referrals and the quality, resource use and outcomes of referrals are lacking [138]

#### Identified deficiencies at the primary-specialty care interface (Table 1)

*D.1. Deficiencies based in clinical decision* making encompass referral errors that pertain to the necessity, appropriateness, timeliness and communication of referrals. Note that the parameters defining the necessity, appropriateness and timeliness of a referral, as well as the information and diagnostics needed to support the referral vary between specialities and are set by current standards of care. Referral errors deviations from the defined parameters that are the result of the diagnostic process, which is probabilistic in nature and implies uncertainty and error. Four types of error were identified:

1. *Unnecessary and inappropriate referrals* are the product of false positives, which occur when the patient is inappropriately/unnecessarily identified as needing a referral to specialty care.
2. *Delayed referrals* are the product of false negatives, which occur when a referral to specialty care is necessary but is not made, resulting in patients not being referred until their condition reaches late stages [22–24].
3. *Triaging errors* result when patients are improperly prioritized based on the severity of their condition.
4. *Communication errors* result when sufficient information and diagnostics are not provided [25–27].

*D.2. Deficiencies based in information management* encompass delays that result due to technological communication breakdowns at the primary-specialist care interface. Traditionally the referral letter has been the principal means of communication and its importance is well recognized [10]. However, unnecessary delays result from the reliance on antiquated communication technologies as a means of transmitting referral requests and subsequent communications and diagnostics between primary and specialty care. Modern information technologies can facilitate improved linkages between primary care providers and specialists [28, 29] and help limit communication delays.

*D.3. Deficiencies based in the management of patient flows between primary and specialty care (Supply and demand management)* encompass both the organization of care pathways traversing the primary-specialty interface and their management. The care pathway is a key determinant of the patient flows from primary to specialty care, dictating the options available to primary care providers and influencing how demand is distributed across specialty care providers and diagnostic service providers. Care pathways are designed to incorporates all relevant factors necessary to provide quality care, but should be as efficient as possible, ensuring that patients receive the appropriate care as quickly as possible. Three types of issues were identified in the management of patient flows between primary and specialty care:

1. *Care pathway management.* Inefficient care pathway structure and resource use hinders patient flows.
2. *Role management (Inter-professional workload imbalance).* Surplus demand placed on specialty care providers, creates bottlenecks within the care pathway (Short term and sustained).
3. *Queue/Referral management (Intra-professional workload imbalance*). Unmanaged distribution of referrals amongst specialty care providers may create an imbalance in specialist utilizations that unnecessarily increases average wait times.

*D.4. Deficiencies in the monitoring of quality-of-care and system performance* result when measurement frameworks, needed to provide decision makers the capability for strategic decision making, are lacking. Quality-of-care/performance improvement is promoted as a core value in the healthcare field, with numerous frameworks developed to measure and track the performance of health systems [30–32].

The impact of these four categories of deficiencies is significant, creating unnecessary delays to appropriate specialty services. At a system level, such deficiencies impact the efficiency of the health system, creating access delays for all referred patients, increased costs and extra strain on care providers. At a patient level, such errors result in a poor patient experience, and potential delays in diagnosis and therapy initiation compromising patient outcomes (e.g. rheumatoid arthritis [33, 34] and chronic kidney disease [35]). A lack of quality-of-care/performance monitoring compounds these issues, making it difficult to identify, evaluate the severity, and prioritise problems that arise at the primary-specialty care interface.

#### Intervention strategies and outcomes (Table 2)

##### I.1. Interventions to reduce referral error

*1. Preventive interventions to reduce the occurrence of false positives, false negatives, triaging errors and communication delays.*

Preventive interventions serve to improve referral quality and reduce the occurrence of referral errors. Referral guidelines [36–47] and education programs [48–52] targeting primary care providers generally serve as the foundation for such interventions. However, guidelines and education alone may be ineffective without feedback opportunities [43, 53–56] and relationship building [57]. Similarly, peer review or shared/supported patient assessment may also reduce rates of unnecessary/inappropriate or delayed referrals and can be implemented via primary care triage clinics [58, 59], multidisciplinary team based diagnosis [14], and peer consult groups [60, 61]. The introduction of a referral process that employs standard referral forms, criteria checklists, diagnostic checklists, scoring systems [14, 62, 63] and assessment tools developed specifically for primary care use [64–72] serves to improve referral quality. Local health services providers should be included in dissemination activities, allowing the intervention to adapt to local circumstances [10, 50, 73].

*2. Screening interventions to detect false positives, false negatives, triaging errors and communication delays.*

Screening interventions serve to catch referral errors that do occur. Reassessing patients on wait lists for need and appropriateness can reduce wait lists [74]. Clerical screening of referrals ensures completeness in terms of needed information and diagnostics [75]. Clinical screening of diagnostics prior to a specialist consult may redirect unnecessary or inappropriate referrals [10, 76]. Screening referrals also allows referrals be triaged based on urgency. Triaging may be conducted through various means, from reviewing clinical findings and diagnostic test results to the development of specific scoring systems [77–80, 81, 82]. Previous reviews of triaging systems concluded that further evidence is required regarding the effectiveness and reliability of triaging [83], and whether triaging improves patient outcomes [84] and waiting times [83, 85]. It is difficult to identify interventions developed to catch type II errors. Since type II error results when a patient should be referred to specialty care but is not, such errors must be caught by the patient themselves. Improved patient awareness may encourage patients to voice their concerns with their primary care provider, or seek a second opinion. Various strategies are outlined for Rheumatoid arthritis [86]. For example, internet based education sites and public awareness campaigns can be used to raise patient awareness. Community screening/outreach programmes provide patients direct access to healthcare professionals and provide alternative means for undiagnosed patients to enter specialist care [87, 88].

##### I.2. Interventions to improve information management- electronic referral systems and electronic medical records to expedite information sharing

Electronic referral systems (eReferrals), supplemented with electronic medical records (EMRs) enable improved information management, improving workflow efficiency and quality-of-care through better linkages between primary and specialty care, improved dialogue, and better coordination of primary and specialty care resources [28, 29, 89]. eReferrals also help facilitate transmision of diagnostics [28, 90], reduce duplicate testing [91] and in some cases enable referral triaging, which can improve the appropriate use of clinic time and significantly improve wait times for patients needing to see specialists [48, 92–94]. While eReferral systems hold great promise, in practice, the successful development and implementation of such systems has been difficult [95]. Barriers include the high cost and extensive strategic planning required for development, privacy and data security, and technical barriers such as interoperability between proprietary EMRs [28]. The benefits of eReferrals may well outweigh these costs but further empirical evidence is required.

##### I.3 Interventions to improve system level patient flows between primary and secondary care

*1. Care pathway management - streamlining*

Patient flows between primary and secondary care may be improved by the removal of unnecessary gatekeeping along the referral pathway, providing direct or open access. Examples include allowing patients to self-refer for follow up on uncertain diagnoses [96], allied health workers to refer directly to specialists [27], and allowing primary care physicians to circumvent preliminary specialist assessments in favour of proceeding straight to advanced diagnostics [26, 97–104, 105, 106] and even surgery [107]. A similar strategy involves specialties running rapid access clinics to triage and provide care to urgent patients [108–111]. The inherent trade-off that comes with providing direct or open access is the potential for increased rate of inappropriate referrals or diagnostic requests. For example, one study found that in an open access system for endoscopic procedures, agreement between GPs and specialists was poor to moderate, with specialists viewing 22.1% of referrals as inappropriate [105].

*2. Human resource management - Scope of practice restructuring.*

In systems constrained by workforce shortages, improved patient flows may be achieved by allocating selected tasks to alternative health care professionals with similar scopes of practice (e.g. [112]). For example, extending the roles of nurses and nurse practitioners in specialty areas such as rheumatology [113], cardiology [114, 115] and oncology [116] can reduce demand for specialists while providing equivalent care with no greater risk of poorer outcomes. Similar roles may be played by allied health care providers such as physiotherapists [117, 118], and primary care physicians [119, 120]. The formation of multi-disciplinary care teams (MDTs) that provide an integrated approach to healthcare and may improve load sharing between medical and allied health professionals who work collaboratively (e.g. [121]). MDTs have become the standard of care in Oncology [122], enabling better patient access to a wide range, but increasingly specialized health care services for disease management [123, 124], but are also thought to be an effective model for chronic diseases requiring complex management strategies [125]. Current evaluations of MDT care focus on clinical benefit to the patient [126, 127] and information on system level outcomes and cost-effectiveness is lacking [128].

*3. Queue/referral management - Centralized intake of referrals to improve access to specialty care.*

Referral management via centralized intake provides a mechanism for manage demand intra-professionally [121, 129–131] and to mitigate the effects of fluctuations in staffing, facility availability, and caseload (emergent, urgent, non-urgent, etc.). Centralized intake has been implemented through dedicated referral management centres, which are tasked with handling all incoming referrals for all specialties, and specialty based clinical assessment clinics. Systematic evaluations of referral management centres are lacking [10, 14]. There is evidence showing speciality based clinics offer improved urgency-based access to specialists [58, 121, 132–134]. While specialty based clinics have demonstrated a decreased wait period from referral to first consult, other wait periods may remain unchanged, such as the wait period from referral to surgery [135, 136]. Specialty triage clinics may also reduce the overall rate of unnecessary and inappropriate referrals, resulting in patients being appropriately diverted from specialist care [137].

##### I.4 Interventions to monitor and improve quality and/or performance - continuous quality improvement

Guavara et al. [138] review performance measures for the specialty referral process, categorizing performance measures into 4 principle domains: 1) referral initiation (reason and rates of referral), 2) accessibility to specialty care 3) coordination of primary and secondary care, and 4) quality (timeliness and satisfaction), with the majority of measures reporting on referral structures. Performance measures for referral processes and outcomes are lacking, and although most of the reviewed measures included assessments of validity, few reported on reliability.

#### Synergies and trade-offs

We end by identifying potential synergies and conflicts among intervention strategies. Recognizing synergies or trade-offs is important when attempting to manage multiple intervention strategies that may seek to achieve common or conflicting objectives [139].

Potential synergies amongst the reviewed interventions were identified. For example, centralized intake of referrals may facilitate the introduction of a standardized referral process, with supporting guidelines, especially if implemented with eReferrals [93, 140–142] improving workflow efficiency by ensuring the completeness of the referral and providing access to relevant information and diagnostics thus hastening the screening of the referral [28, 90]. In addition, standardized referral forms ensure the patient’s information, history, physical exams, laboratory tests and urgency are communicated to the specialist [143–145], improving triaging. Centralized intake may also facilitate the introduction of both preventive and screening interventions to reduce inappropriate/unnecessary referrals. When coupled with EMRs, referrals can be clinically screened, reducing the need for face-to-face assessment, instead allowing referrals and diagnostics to be reviewed remotely [48, 93, 94]. Triaging may be coupled with rapid access services to reduce wait times between referral and patient assessment [110].

Identifying potential conflicts between intervention strategies requires an understanding of the mechanisms of causality that explain why deficiencies arise, and how they are corrected via intervention. For example, our discussion of referral errors (false positives and false negatives) and the corresponding corrective interventions would not be complete without acknowledging that in many cases a trade-off will need to be made between inappropriate /unnecessary referrals and delayed referrals. Indeed, a decreased specificity and increased sensitivity will result in fewer false negatives but increased false positives, which will increase the demand for specialty services. Alternatively, an increased specificity and decreased sensitivity will result in fewer false positives but an increased number of false negatives which will increase the number of patients remaining undiagnosed and improperly managed. Interventions are needed to not only improve the sensitivity and/or specificity of the decision process, but also to optimise the balance between the sensitivity and specificity. Similarly, interventions designed to reduce or catch referral errors (sometimes called gatekeeping) may be in conflict with accessibility. For example, screening interventions to reduce rates of unnecessary and inappropriate and/or delayed referrals may introduce additional waiting periods. Such processes must be effective in redirecting inappropriate referrals in order to offset additional delays and costs that are introduced. If the false positive/negative error rates are sufficiently low, then it may be of benefit to move towards a more direct, or open access, referral system that eliminates one or more screening steps along the traditional referral pathway. Conversely, removal of screening processes to improve access times to diagnostics and specialty services may increase the frequency of inappropriate/unnecessary referral to these services, increasing wait times for all patients (appropriately and inappropriately referred). Finally, balance between system, provider, and patient perspectives is always needed. For example, when assessing referral management strategies such as centralized intake, caution is required not to limit focus purely to system efficiency. Both primary and specialist provider participation is crucial for effective centralized intake systems. Reasons for non-participation include loss of autonomy, loss of primary-specialist care relationships, and loss of control over case-loads [135]. Patients may prefer the option to have input into the scheduling of appointments [146], but appropriateness may decrease [147]. Interestingly our search revealed little if any discussion regarding the strategic balancing of these effects.

## Discussion

There is a large literature pertaining to interventions that impact the necessity, appropriateness, timeliness and communication of patient transitions across the primary-specialty care interface. The literature is broad and can be difficult to navigate due to complexity of the primary-specialty care interface, the breadth of potential deficiencies, and the equal breadth of interventions developed to correct them. As such, we present a narrative review that describes linkages between deficiencies at the primary-specialty care interface, which impact access to specialty services for non-emergent patients, with interventions and subsequent effects.

This review was focussed only on deficiencies at the primary-specialty care interface that impact access to specialty services to address our objective on these specific problems. The review does not consider deficiencies at the primary-specialty care interface that impact the broader patient and service provider experience. While improving access to specialty care is a critical issue in the delivery of health services, more broadly, health system performance is measured in terms of quality of care criteria in multiple dimensions. Improving access to care must be done in the context of the broader objective of improving the overall quality of care provided to patients suffering from chronic diseases.

### Limitations

This review is unlikely to be complete, especially given the broad scope of our topic. The literature on health services interventions applied at the primary-specialty care interface is vast, spanning many fields from clinical practice to economics and operations research. Inevitably there are bodies of work that will be revealed to have been left out. For example, the omission of CINAHL in our search strategy may have resulted in missed studies pertaining to the roles and impact of allied health care workers and nurse practitioners in reducing wait times for specialist services when the service the patient required could be provided by a nurse practitioner or an allied health care provider. Moreover, as the primary-specialty care interface continues to evolve, new problems will inevitably arise and future research will produce new innovative health system interventions.

We did not summarise the context and assess the quality of each study. Our aim and focus were to note that from a system perspective, the literature is fragmented. Specifically, the studies reviewed were generally highly context specific; developed to document improvements to in-use, specialty specific, referral systems experiencing specific difficulties (e.g. long wait times). Such an approach does not lend itself to establish the effectiveness of the intervention in the broader health systems context. Consequently, it is not clear if the available evidence pertaining to intervention effectiveness can be taken out of context. The reviewed studies were not developed to demonstrate intervention effectiveness in variety of applications and contexts.

### Value

The key strength of this review is its emphasis on the identification and categorization of deficiencies in the primary-specialty care interface by cause. Within the literature, interventions are commonly framed in terms the practical actions taken to create improvements in system performance as measured through specific outcomes such as reduced wait times between referral and consult. However, simply detailing an intervention’s actions and outcomes without describing the deficiencies the intervention is designed to correct can lead to potential mis-application. The potential issue here is one of logical verification; the logic that supports the adaptation and implementation of the reviewed interventions to new contexts can only be verified through an understanding as to why the performance of the primary-specialty care interface is failing in the first place. The intention is that such a causal approach will facilitate the development of complex integrated health system interventions that consist of multiple coordinated intervention components, managing different shortfalls in the referral system that arise at the primary-specialty care interface. The categorization serves as a necessary first step to facilitate the development and evaluation of such complex interventions. The practical outcome is the creation of a resource for health care organizations seeking to optimize the primary care/specialty care interface to improve access to specialty care for non-emergent patients from a systems perspective.

## Conclusion

The results of this review demonstrate the breadth of deficiencies that impact access to specialty services for non-emergent patients via primary-specialty care interface and an equal breadth of corrective interventions. Although interventions developed to improve the referral process at the primary-specialty care interface hold great promise, much work remains to better understand the potential utility of such interventions. Deficiencies that limit access to specialty care for non-emergency patients, as outlined here, arise independent of context. As such, general intervention strategies that can be adapted and applied in broad range of contexts are needed.

## Additional file

Additional file 1: Search strategy. Details of the search strategy used. (DOCX 13 kb)

## Abbreviations

EMRs: Electronic medical records
