# Supplementary material for: A categorisation of problems and solutions to improve patient referrals from primary to specialty care
Source: BMC Health Serv Res. 2018 Dec 20;18:986. doi: 10.1186/s12913-018-3745-y (PMC6302393; doi:10.1186/s12913-018-3745-y)
Supplement: Supplementary file 1 — Search strategy. Details of the search strategy used. (DOCX 13 kb) [file 12913_2018_3745_MOESM1_ESM.docx]

**Appendix - Search Strategy**

**** 1. Limit by area of practice****

1. exp surgical procedures, elective/

2. exp specialization/

3. (elective service* or elective surg* or scheduled service* or scheduled surg* or elective procedure or non-emergenc* procedure* or day surger* or non-emergen* surger* or routine surg* or routine procedure* or (outpatient adj3 (surger* or procedur*)) or (out-patient adj3 (surger* or procedur*))).tw.

4. (secondar* care or special* care).tw.

5. 1 or 2 or 3 or 4

**** 2. Limit by research area (Health services Research –improvement wrt specific dimension or outcome)****

6. exp health services accessibility/

7. exp Comparative Effectiveness Research/

8. exp Efficiency, Organizational/ or exp Efficiency/

9. exp Patient Safety/ or exp Safety management/

10. exp benchmarking/

12. (Cost* or efficien* or effect* or outcome* or safe* or appropriate* or mis-use or over-use or inappropriate* or access* or allocat* or mis-allocat* or patient flow*).tw.

12. 6 or 7 or 8 or 9 or 10 or 11

**** 3. Search known management strategies ****

13. exp referral/ and consultation/

14. Patient care team/

15. resource allocation/ or health planning/

16. triage/

17. exp appointments/ and schedules/

18. exp waiting lists/

19. (multi-dis* team or multi* care).tw.

20. (wait* list* manag* or queu* manag* or single-entry or central* intake or common intake or single point-of-entr* or pooled or pooling or triag* or referral* manag*).tw.

21. 14 or 15 or 16 or 17 or 18 or 19 or 20

**** Combine 1, 2, and 3 ****

22. 6 and 13 and 21
